# Dataset on occupational health conditions and safety practices among welders in Bangladesh

**DOI:** 10.1016/j.dib.2025.112151

**Published:** 2025-10-08

**Authors:** Rakhi Moni Saha, Fatama Binta Rafiq, Plabon Kumar Saha, Imran Mahmud

**Affiliations:** a Department of Software Engineering, Daffodil International University, Dhaka, Bangladesh; b Department of Computer Science and Engineering, American International University, Bangladesh

**Keywords:** Welding, Occupational health, Eye injuries, Respiratory hazards, Safety practices, Bangladesh, Machine learning, Dataset

## Abstract

This data article provides a dataset on occupational health and safety of welders in Bangladesh regarding eye injury, respiratory disorder, and associated risk factors. The data, collected on a pre-tested structured questionnaire, consist of demographic information (age: 10–77 years, median ∼30 years; working experience: 1–50 years), work environment features (working time per day, type of welding, use of protective equipment), and self-reported health consequences (e.g., burning eyes, headache after work, breathlessness). The data set includes raw data, symptom frequency summary tables, a health symptoms by lifestyle element correlation matrix, and a sample machine learning program for predicting post-work headaches. All raw data are placed in the public domain through the Mendeley Data repository for reuse. No conclusions were drawn in this paper; instead, we present the data and how it may be utilized to allow for further studies on occupational health hazards and prevention measures among welders.

Specifications Table

**Table:** 

Subject	Public Health / Occupational Health and Safety
Specific subject area	Health risks and safety practices among welders in Bangladesh
Type of data	Table; Chart; Figure (survey dataset in spreadsheet; graphical figures)
How data were acquired	In-person interviews with a set questionnaire; responses were audio-recorded on site at welding workshops (with participants' consent). There was no special equipment needed beyond standard survey instruments.
Data Format	Raw (primary survey responses in an Excel/CSV file); Analyzed (summary tables, figures, and derived variables included)
Parameters for data collection	Welders across all levels of experience from small workshop settings were interviewed across five districts of Bangladesh. Full-time welders and multipurpose metal workers were interviewed. Work history, the welding process (arc/gas), the use of protective tools, and the prevalence of current compared to previous health complaints (ocular and respiratory symptoms) were the main elements of data collection.
Description of data collection	Data was gathered in the field during 2022. Participants were recruited from Meherpur, Rajshahi, Sirajganj, Jamalpur, and Mymensingh district workshops. After being obtained with informed consent, the questionnaire was filled in, for demographic data (age, gender), employment details (years experienced, hours worked per day, how welds are performed, training received, use of personal protection equipment), and self-reported health ailments (e.g. any current breathing problem, any problem with eyesight, how often the eyes irritate or burn, facial burns, headache after leaving work, difficulty sleeping). It was documented and summarized within a spreadsheet. The survey also documented if the worker previously had breathing or vision problems prior to beginning welding, in order to differentiate occupational-related health effects.
Data source location	Institution: Daffodil International University (data collection conducted in field locations) City/Region: Meherpur (23.75°N, 88.70°E); Rajshahi (24.37°N, 88.60°E); Sirajganj (24.46°N, 89.70°E); Jamalpur (24.92°N, 89.96°E); Mymensingh (24.75°N, 90.40°E) – Bangladesh.
Data accessibility	Repository name: Mendeley Data [1] Data identification number: 10.17632/hmd8b3nbff.3 Direct URL to data: https://data.mendeley.com/datasets/hmd8b3nbff/3
Related research article	N/A

## 1 Value of the Data

• This dataset is novel, structured data on occupational health risk among welders within a low-resource setting. It quantifies the prevalence of complaints of eye burning, difficulty breathing, and headache, along with data regarding working conditions and the use of PPE. Researchers can examine how variables like experience, work duration, and safety tool use correlate with complaints of health issues, creating a rich epidemiological reference point for policy and intervention planning in Bangladesh.
• The data can be of use to public health researchers, occupational hygienists, policymakers, and safety engineers. It can help in risk assessment for diseases such as "arc eye" or sickness due to fumes and can inform targeted training on PPE and health screening. It can be used by regulatory agencies to determine workplace safety standards for informal sectors too.
• Statistical analysis of correlation (e.g., PPE use vs. headaches) or training predictive models to predict risk can be conducted by users. The given example and correlation matrix show possible exploratory and predictive paths. Data may also inspire longitudinal studies of causal pathways between exposure and outcome.
• This is one of the oldest open-access health datasets in the context of welding in Bangladesh. It has diverse geographic and occupation-type coverage, pre- and post-state of health, and behavior-driven data like smoking. Its ease of access makes it an invaluable tool for academic and applied research across the globe. Although collected in Bangladesh, the dataset serves as a benchmark for other low-resource environments with similar informal welding sectors, facilitating cross-country comparisons and adaptation of safety interventions.

## 2 Background

Welding is an important industrial process prevalent in the construction, manufacturing, and repair industries globally [2]. Welders are exposed to various occupational hazards like ultraviolet radiation, heat, welding fumes, airborne particulate matter, and metal oxides that may lead to acute and chronic diseases. General health symptoms reported by welders include respiratory diseases, impaired vision, burns on the skin, headaches, and sleep disturbances. Occupational health risks have been reported to exist in spite of the current occupational health and safety regulations, particularly among developing nations[3,4].

It is consumed at a rapid rate in Bangladesh, where there is industrialization going on. Welding is of utmost significance in the formal and informal economy in Bangladesh. There are few welder occupational health studies for any welder, and also there are no longitudinal data. The welders in Bangladesh primarily work in small workshops or in the informal economy without protective equipment and proper training. It is poor occupational health control within the workplace, which exposes them to occupational health risks and results in unnecessary disease and injury [5].

Here, the necessity for quantitative, accurate information regarding both occupational health status and the working environment of Bangladeshi welders becomes clear. Such information would be extremely useful in making clear the relationship among welding methods, protection measures, and health outcome [6,7]. The data set presented here fulfills this requirement by offering systematically collected, true facts about a representative sample of welders in several districts of Bangladesh. Such data can prove to be an important tool for occupational health experts, policymakers, and researchers alike in their efforts to raise the occupational safety levels and minimize health risks in welding occupations. This dataset offers a localized perspective with limited national representativeness.

## 3 Data Description

The dataset is available in a Microsoft Excel file titled ”Dataset_of_Welder_health_Survey_Bangladesh.xlsx” and contains one main sheet. We have collected data from 335 welders from Bangladesh around five places: Meherpur, Rajshahi, Sirajganj, Jamalpur, and Mymensingh district workshops. The following is the structure of the dataset, which explains the columns and their significance (Table 1).

**Table 1:** Overview of the columns in the datasets.

**Column Name**	**Data Type**	**Description**	**Example Values**
timestamp	Datetime	Date and time when the survey was conducted	8/3/2022 0:07
Age	Numeric	Age of the respondent (years)	23
Gender	Category	Gender of respondent (all male)	Male
job_type	Category	Type of job (Full-time welder or Multipurpose worker)	Full-time welder
workshop_name	Text	Name of welding workshop	[Various unique names]
work_experience_years	Numeric	Total welding experience (years)	5, 10, 15
daily_work_hours	Numeric	Average number of hours worked daily	6–10 (typically ∼8.4)
welding_training	Category	Formal training received (Yes/No)	Yes, No
welding_type	Category	Type of welding primarily used (Arc, Gas, Both)	Arc, Gas
gas_welding_years	Numeric	Experience specifically with gas welding (years)	0–15
arc_welding_years	Numeric	Experience specifically with arc welding (years)	0–20
welding_process	Category	Specific welding techniques used	Manual metal arc, MIG
machine_type	Category	Type of welding machine used	Transformer, Inverter
machine_range	Category	Welding machine current range	<120 A, 120–180 A, >180 A
uses_safety_tools	Category	Regular PPE used (Glass, Helmet, Gloves, Apron, None)	Glass, Helmet
smoker	Category	Smoking status of respondent (Yes/No)	Yes, No
prior_breathing_issues	Category	Had respiratory issues before welding (Yes/No)	Yes, No
current_breathing_issues	Category	Currently has respiratory issues (Yes/No)	Yes, No
prior_eyesight_issues	Category	Had eyesight issues before welding (Yes/No)	Yes, No
current_eyesight_issues	Category	Currently has eyesight issues (Yes/No)	Yes, No
eye_burning_issue_sleep_time	Category	Experiences eye irritation at bedtime (Yes/No)	Yes, No
face_burn	Category	Experienced facial burns from welding (Yes/No)	Yes, No
headache_after_work	Category	Experienced headaches post-welding (Yes/No)	Yes, No
sleeping_disorder	Category	Experiences sleep-related issues (Yes/No)	Yes, No
photo_consent	Category	Consent for using photos publicly (Yes/No)	Yes, No

**Structure of the File:** filename=Dataset_of_Welder_health_Survey_Bangladesh.xlsx

**Data Type:** Tabular data containing raw and calculated metrics.

To make it clear, categorical variables like welding_training, smoker, and prior_breathing_issues are shown as either binary or multiple-choice answers. For binary variables, ‘Yes’ is marked as 1 and ‘No’ as 0. The original labels are kept in the raw data so it's easier to understand. Some variables, like gas_welding_years or arc_welding_years, might have empty spaces, which means the information wasn't provided or doesn't apply. We didn't fill in those missing values to keep the data as it was. Users should decide how to handle missing data based on their own needs, like using averages or just leaving them out. Overall, there aren’t many missing data points.

### 3.1 Folder/subfolder structure for dataset repository

1. **Root Folder**
  a. README.txt: A detailed description of the dataset, including purpose, methods, and data format.
  b. Dataset_of_Welder_Health_Survey.xlsx: The main dataset containing all variables.
  c. Python Code: The data visualization codes have also been included.
2. **Subfolder: Figures**
  a. Includes illustrative figures of data distribution or trends (such as histograms or scatter plots, pair plot, box plot, or correlation).

### 3.2 Visualization

**Fig. 1:**
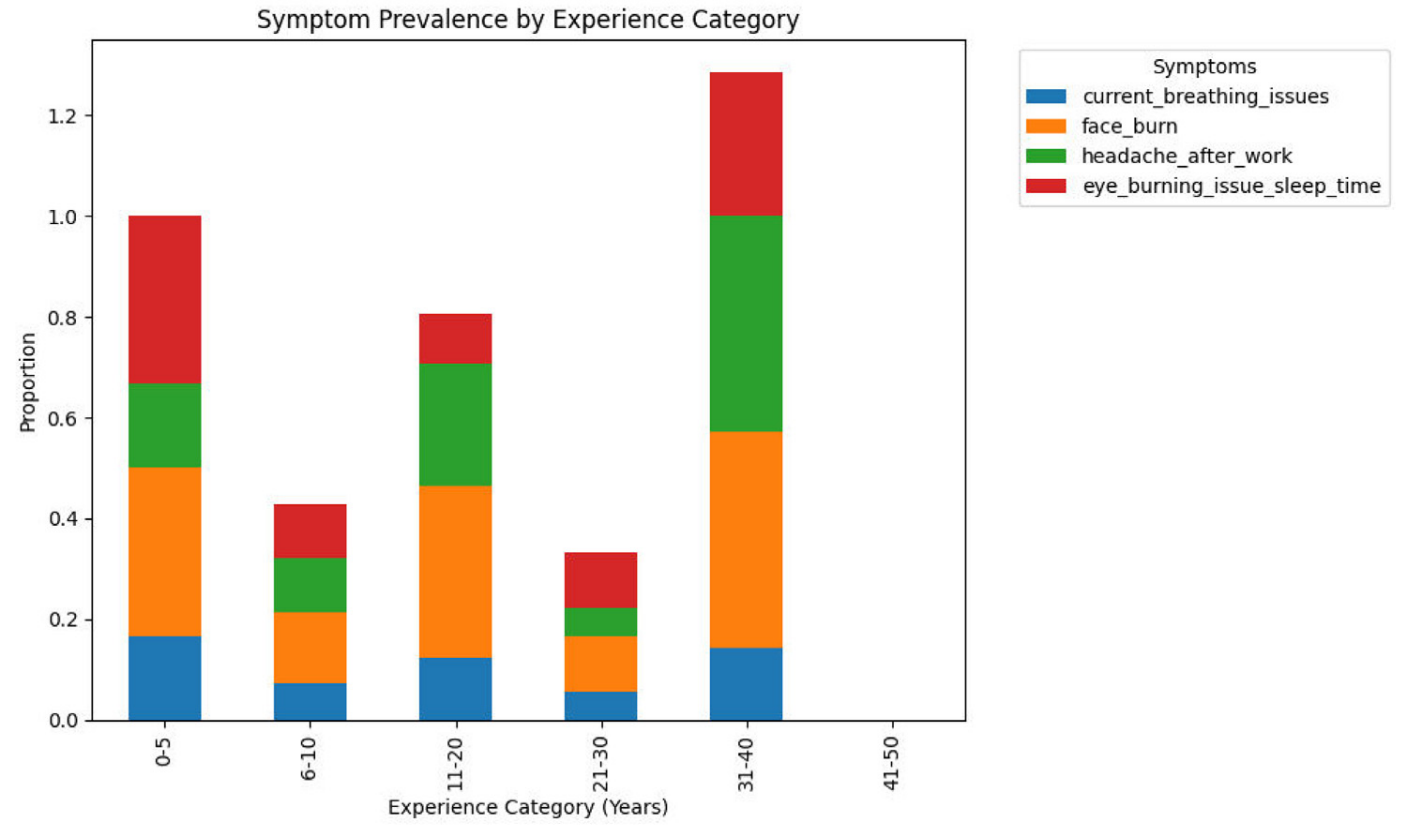
Prevalence of symptoms by welding experience category.

Fig. 1 shows the percent of welders reporting various health-related symptoms across various categories of welding experience (0–5, 6–10, 11–20, 21–30, and 31–40 years of experience). The colored portion of a bar is the percentage of welders within an experience group who have noted a given symptom: current breathlessness, facial burns, headache after work, or eye irritation disturbing sleep. The 21–30 years of experience welders reported the lowest overall incidence of symptoms, whereas those in the most recent (0–5 years) and most experienced (31–40 years) groups reported greater overall symptom rates.

**Fig. 2:**
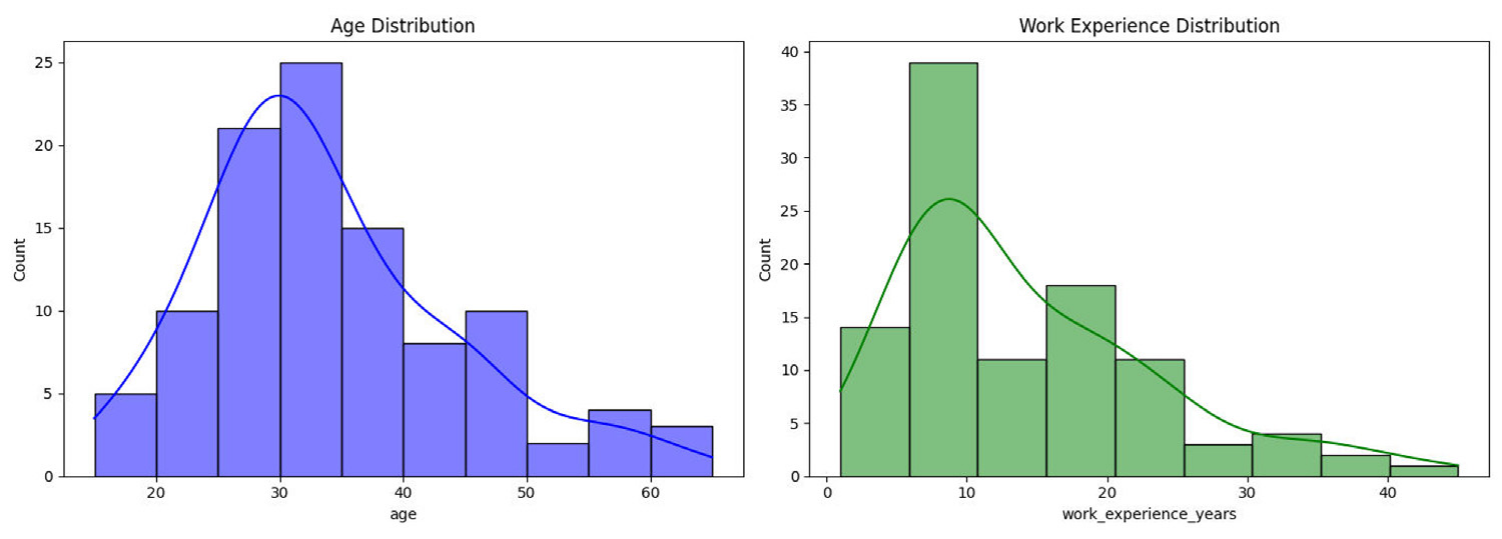
Distribution of the ages of welders and years of welding work experience.

The Fig. 2 presents distributions of the ages of welders (left panel, in blue) and years of welding work experience (right panel, in green). Each panel is a histogram with a smoothed density curve overlaid. The age distribution indicates that most welders in this data are in their late twenties to mid-thirties, with relatively fewer welders at older ages. The work experience is distributed in a right-skewed manner, reaching a peak between 8 and 12 years of experience; the majority of the welders possess between 5 and 15 years of welding experience, with not many exceeding 20 years in the trade.

**Fig. 3:**
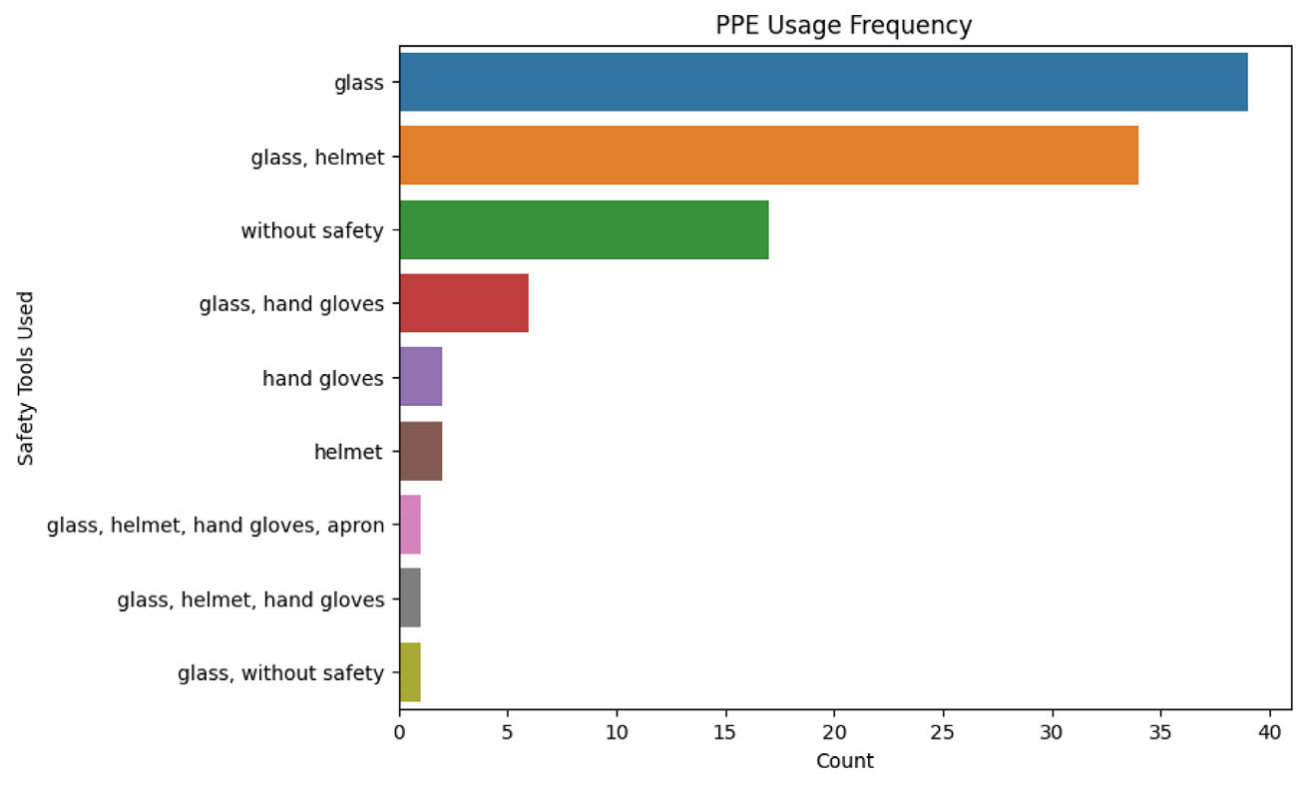
Frequency of use of personal protective equipment (PPE) by welders.

Fig. 3 illustrates the frequency with which different combinations of PPE are utilized by the welders. Each bar represents a specific pattern of PPE usage and the number of welders who indicated that practice. The most common practice is the use of safety glasses alone, followed by the use of safety glasses and a welding helmet. A large number of welders indicated no usage of protective gear whatsoever, pointing to a primary safety deficiency. On the other hand, a very small number of welders use the full PPE (glasses, helmet, hand gloves, and apron) while working.

**Fig. 4:**
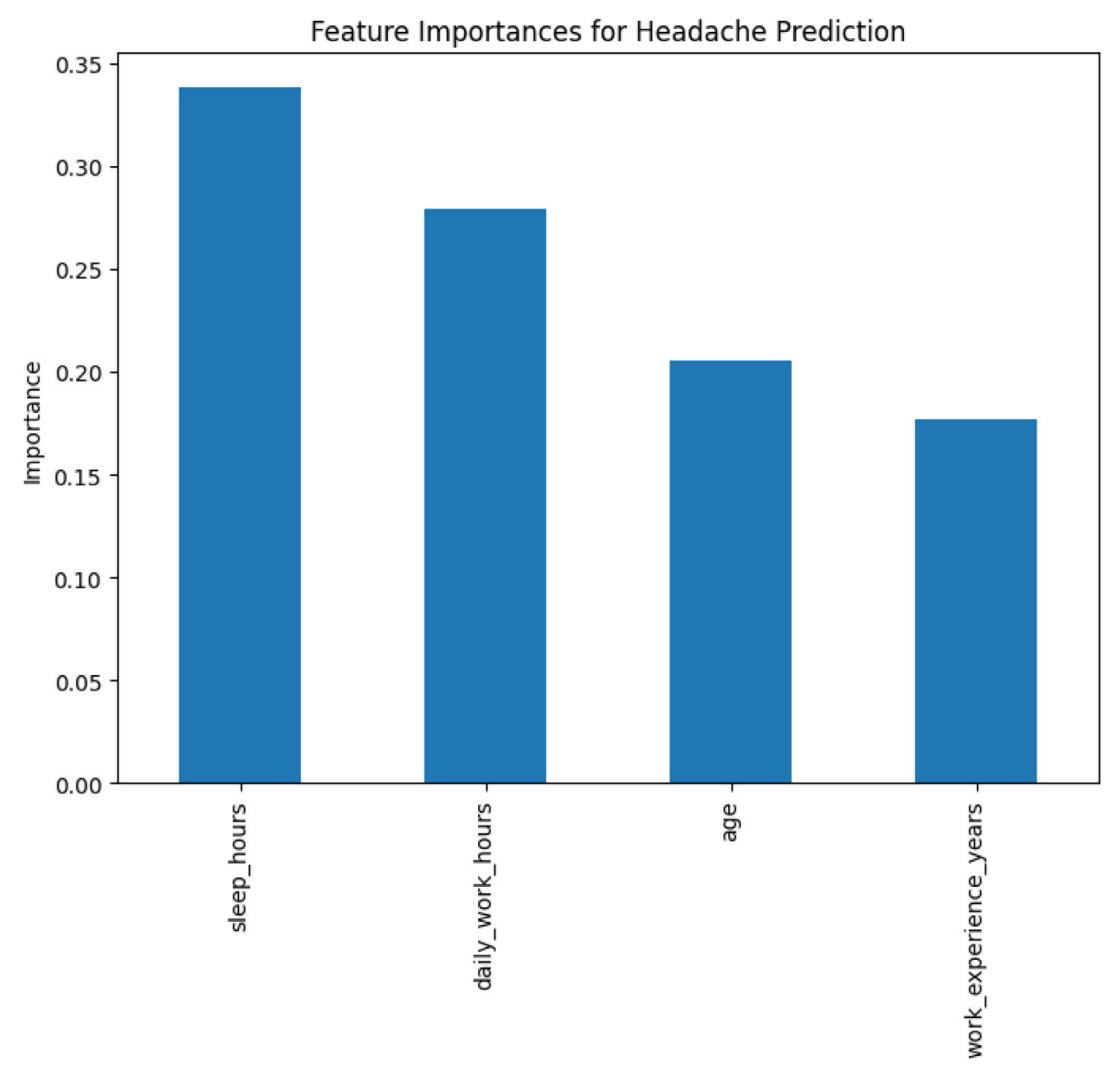
Key predictive features for post-work headache (Random Forest classifier).

Fig. 4 shows the relative importance of the most predictive features for whether a welder experiences headaches after work, based on a Random Forest classification model. Among the variables considered, the average sleeping hours per day ("sleep hours") is the most significant predictor, followed by working hours per day. Age and total years of work experience carry slightly lower importance weights in the model. All of these variables have non-zero importance, indicating that each of them contributes some part to headache occurrence prediction, with enough sleep and reasonable working hours being the most contributing factors associated with a reduction in post-work headache risk in this experiment.

**Fig. 5:**
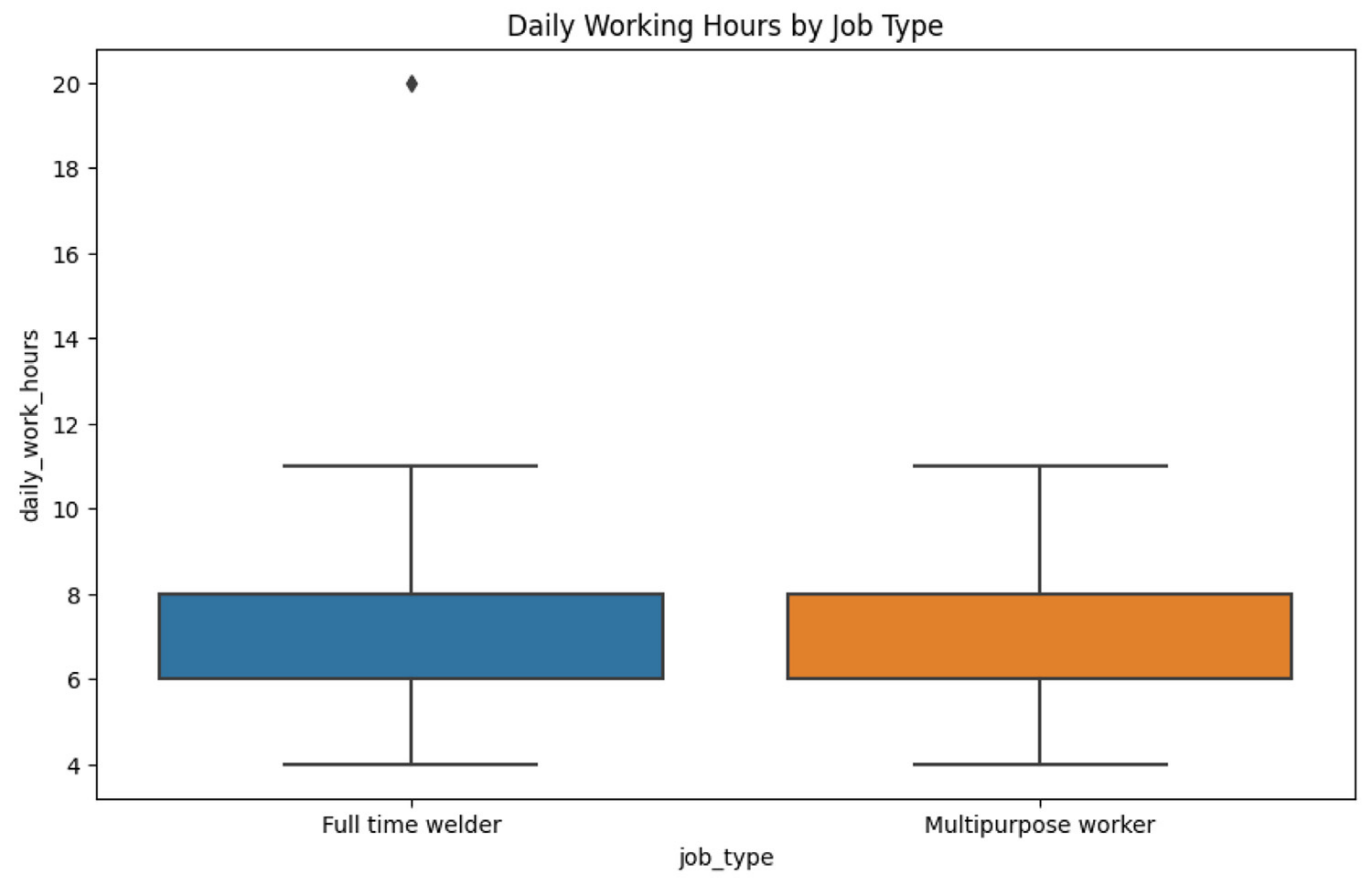
Comparison of daily working hours across job types.

Fig. 5 compares the distribution of self-reported typical daily work hours for full-time welders and multipurpose workers (workers who perform welding along with other tasks). Each box shows the median (center line) and interquartile range (box boundaries) for hours worked per day, with whiskers to the minimum and maximum values (excluding outliers, which are plotted as individual points). The median working day duration is similar for both groups, between 7 and 8 h. Full-time welders do have a wider range of working hours and more variation; there were some extreme values where full-time welders had very long workdays (outliers up to ∼19–20 h), which were not present in the multipurpose workers.

**Fig. 6:**
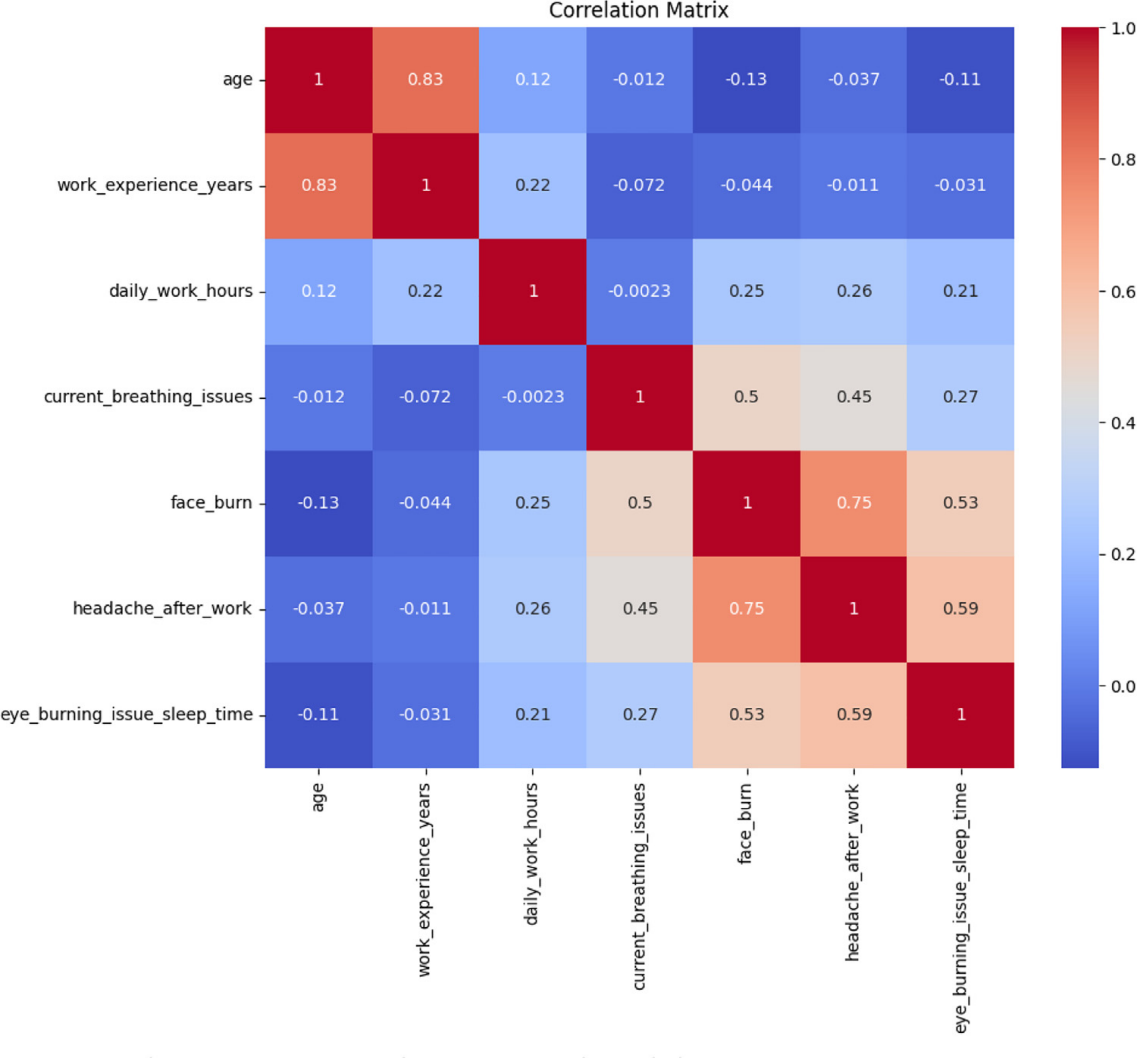
Correlation matrix between health indicators and demographics.

Fig. 6 shows Pearson correlation coefficients between selected demographic variables (age, years of work experience, hours of work per day) and outcome health variables (current breathlessness, face burn, headache after work, and eye irritation disturbing sleep). The darkness of the color (red for positive correlations, blue for negative) is proportional to the size of the correlation (numerical values are printed in each cell). As might be anticipated, work experience is very strongly positively correlated with age (*r* ≈ 0.83). There are also moderate positive correlations among the health symptoms - for example, face burns and headache after work are correlated with each other at about 0.75, indicating that these issues tend to go together.

**Fig. 7:**
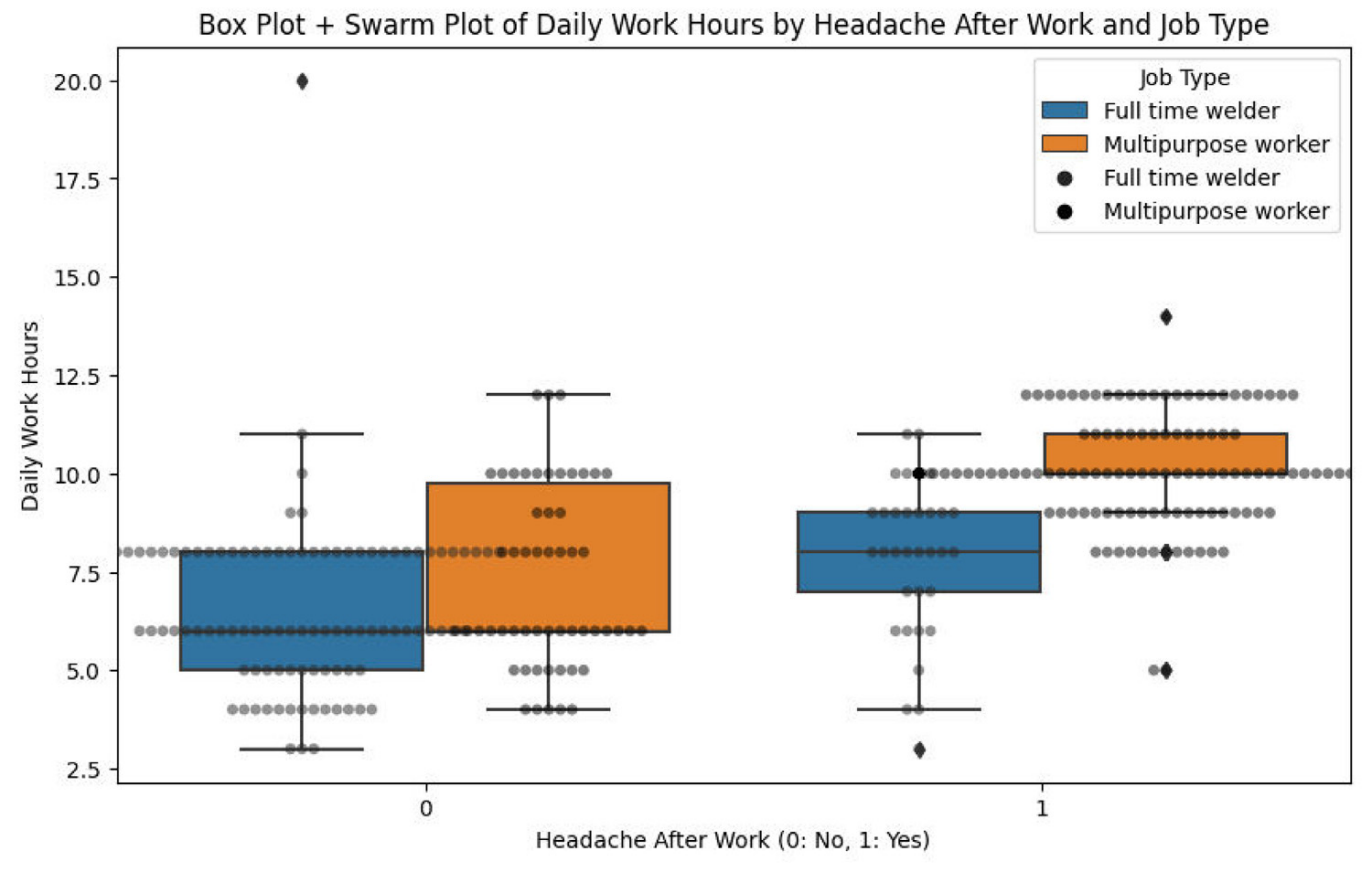
Box plot + swarm plot of daily work hours by headache after work and job type.

Fig. 7 is a straightforward representation of the correlation of working hours per day, incidence of headaches, and category of work among 335 welders surveyed in Bangladesh, providing important information regarding occupational health risks. The x-axis separates workers according to headache incidence (0 = no, 1 = yes) after work, and the y-axis indicates working hours per day from 2.5 to 20 h. Box plots, colored by employment type (blue for full-time employees classified as welders, orange for multipurpose workers), indicate interquartile range (IQR) and median, whiskers to minimum and maximum values excluding outliers (marked as diamonds), and an overlay swarm plot indicates distribution of individual data points. For welders with no headaches (0), there is a median of about 7.5 h (IQR: 6.5–9 h) with outliers to 12.5 h for full-time welders, and a median of about 8 h (IQR: 7–10 h) with fewer outliers for multipurpose workers. On the other hand, headache welders (1) have larger medians, 8 h for full-time welders (IQR: 7–12 h) with an outlier of 17.5 h, and 9 h for multipurpose workers (IQR: 8–12 h) with an outlier of 15 h, demonstrating a clear relationship between the longer workday and the occurrence of headaches, particularly the full-time welders with greater variability and extremity of work hours. This article brings to light the need for intervention to lower health risks linked to long working hours among these individuals.

Generally, the data indicate substantial diversity in welders' work conditions and their associated health implications. The figures above highlight some of the key findings: certain health symptoms are prevalent among welders, especially those at the extremes of experience (new or highly experienced workers); a high proportion of welders are not using adequate protective gear; and lifestyle or work factors including sleep deprivation and long working hours are likely to be causing headaches and possibly other illnesses. These findings highlight the necessity for occupational health interventions for Bangladeshi welders.

## 4 Experimental Design, Materials, and Methods

### 4.1 Survey design and participant recruitment

Data were collected using a cross-sectional survey with a structured questionnaire on welder demographics, working conditions, and health. The surveys were conducted face-to-face by researchers or trained interviewers in welding workshops in Rajshahi and Sirajganj, Bangladesh, with supplemental responses from Jamalpur, Mymensingh, and Meherpur. Convenience and purposive sampling were used; the welders were approached at work and gave verbal consent voluntarily (see Ethics Statement). Anonymity was protected by not collecting personal identifiers. The only criteria for inclusion were being a welder employed full-time or part-time and agreeing to an interview. All 335 approached welders provided complete responses. *Questionnaire pre-tested on 10 welders for clarity.*

### 4.2 Data collection process

1. **Demographics and experience:** Gender, age, welder experience, and whether formally trained. Formal training was enjoyed by only ∼7 % (23 welders), who had otherwise been trained informally or on the job.
2. **Job features:** Full-time vs. multipurpose worker employment type, employment type of welding practices used (arc, gas, or both), and work hours per day. Approximately 70 % of them did predominantly electric arc welding; 30 % did arc and gas welding.
3. **Safety equipment and procedures:** Use of safety equipment/PPE (glasses, gloves, helmets, aprons, or none). Respondents could have more than one answer and these were clustered for analysis.
4. **Health history and symptoms:** Underlying and present health grievances, i.e., breathing and vision problems, face burns, headache upon returning from work, eye irritation affecting sleep, and sleep diseases, were mentioned by the participants. Additionally, smoking status (∼46 % smokers) was enrolled in order to control respiratory health confounders.

Bengali interviews lasted between 15 and 20 min on average. The answers were captured digitally in real-time utilizing smartphone/tablet, timed, and automatically compiled into an Excel dataset.

### 4.3 Data preprocessing

Raw data were inspected for completeness and consistency. Categorical response (e.g., health symptoms) were recoded to binary (Yes=1, No=0) in analysis. Minimal text cleaning (especially PPE responses) was undertaken for consistency. Out-of-range data (e.g., 20 daily working hours, age 10) were retained for realism, and users were left to choose data removal. No entries were removed, and no imputation had to be undertaken because complete responses were available.

### 4.4 Data analysis and visualization

Analysis was done in a Python Jupyter Notebook provided with the package data. Libraries used were pandas, NumPy, matplotlib/seaborn, and scikit-learn. Analytical steps were:

• **Descriptive statistics:** Frequencies, percentages, and summary statistics for variables (e.g., mean age, distribution of experience, use of PPE, prevalence of health symptoms).
• **Visualization:** Histograms (Fig. 1) produced, boxplots (Fig. 2), frequency bar plots of PPE (Fig. 3), correlation matrix heatmap (Fig. 4), incidence of headache vs. working hours (Fig. 5), symptom prevalence by experience level (Fig. 7).
• **Correlation analysis:** Pearson correlation matrix computed for selected numeric and binary variables (Fig. 4), quantifying the relationships (e.g., checking strong correlation between headache and daily hours).
• **Predictive modeling:** A random forest classifier was used to predict post-work headaches with predictors including age, experience, daily work hours, smoking status, training status, sleep hours, and PPE use. Feature importance was plotted (Fig. 6), with daily work hours emerging as the most predictive factor.
• **Validation and consistency checks:** Simple checks confirmed category totals equaled each other, and binary transformations accurately represented original responses.

Each analysis notebook step is reproducible, traceable, and amenable to secondary analysis (e.g., stratified analyses or other predictive modeling). Raw data and code shared are an extensive toolkit for secondary exploration of the dataset. *Jupyter Notebook includes reproducible code with requirements.txt.*

### 4.5 Sample demonstration

The Table 2 shows that model was good with 84 % accuracy and balanced F1-score of 0.85, reflecting consistent prediction for both classes. Precision and recall findings suggest that the model is capable of predicting both instances (headache after work and no headache) reasonably well at the cost of some slight negatives for false positive prevention. Cross-validation tested the stability of the model, and the average F1-score was found to be 0.82, although variation between folds was observed.

**Table 2:** Model performance results.

**Category**	**Metric**	**Value**
**Class Balance**	Class 1 (Yes)	54.9 %
	Class 0 (No)	45.1 %
**Overall Performance**	Accuracy	0.84
	Mean F1-Score	0.85
**Class 0 (No Headache)**	Precision	0.79
	Recall	0.87
	F1-Score	0.83
**Class 1 (Headache)**	Precision	0.88
	Recall	0.81
	F1-Score	0.85
**Cross-Validation**	Mean CV F1	0.82 ± 0.17
	Range	0.54 – 0.96

**Fig. 8:**
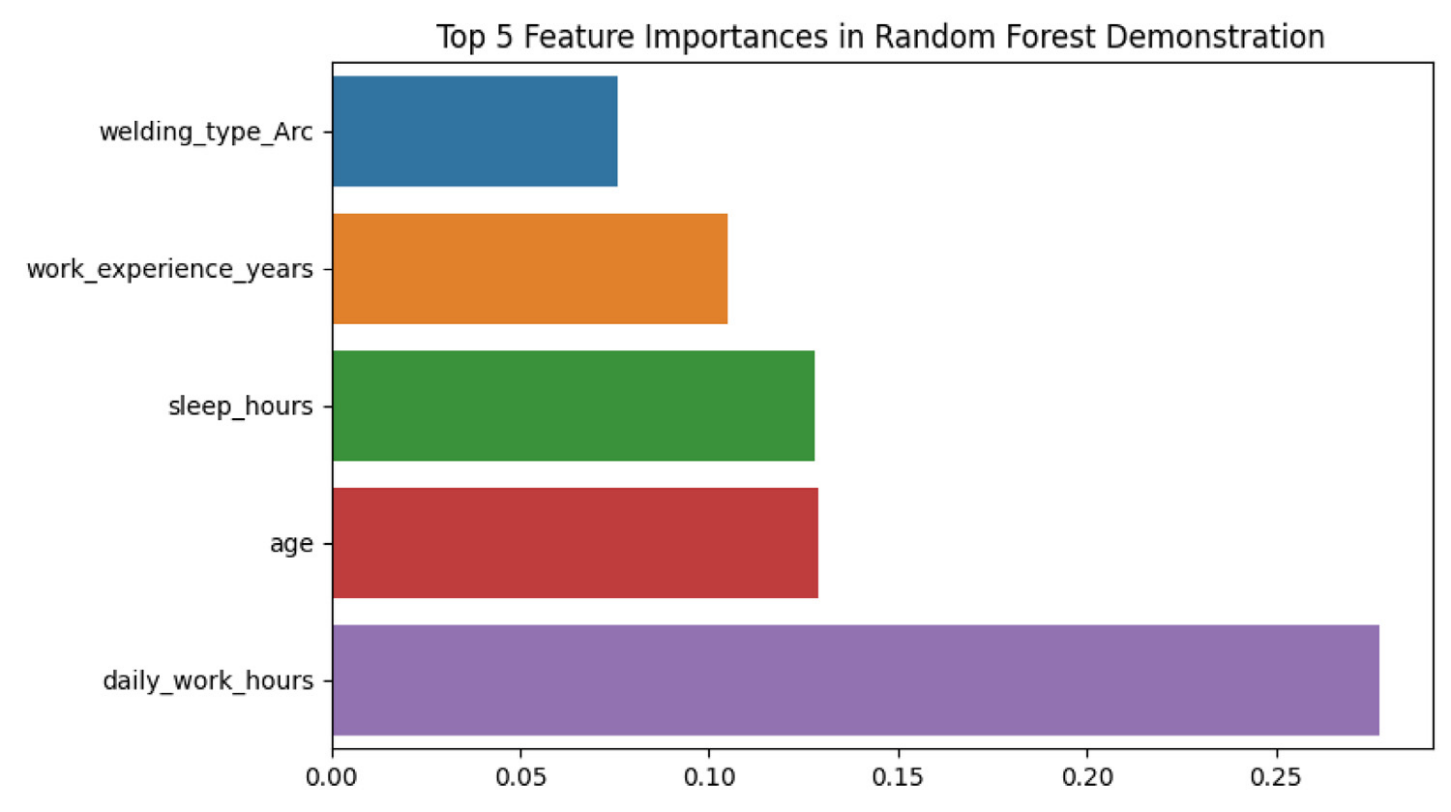
Top predictors of post-work headache (random forest).

Fig. 8 represents a plot of feature importance from a Random Forest model predicting whether welders experience headaches after work. The output suggests that daily working hours are most predictive, followed by average sleeping hours and age. Years of work experience are also beneficial, though to a lesser degree, and welding type is the least influential among the leading features. These findings demonstrate that long working hours and sleep deficiency are the most important risk factors for post-work headache, highlighting the importance of the regulation of working hours and rest.

**Fig. 9:**
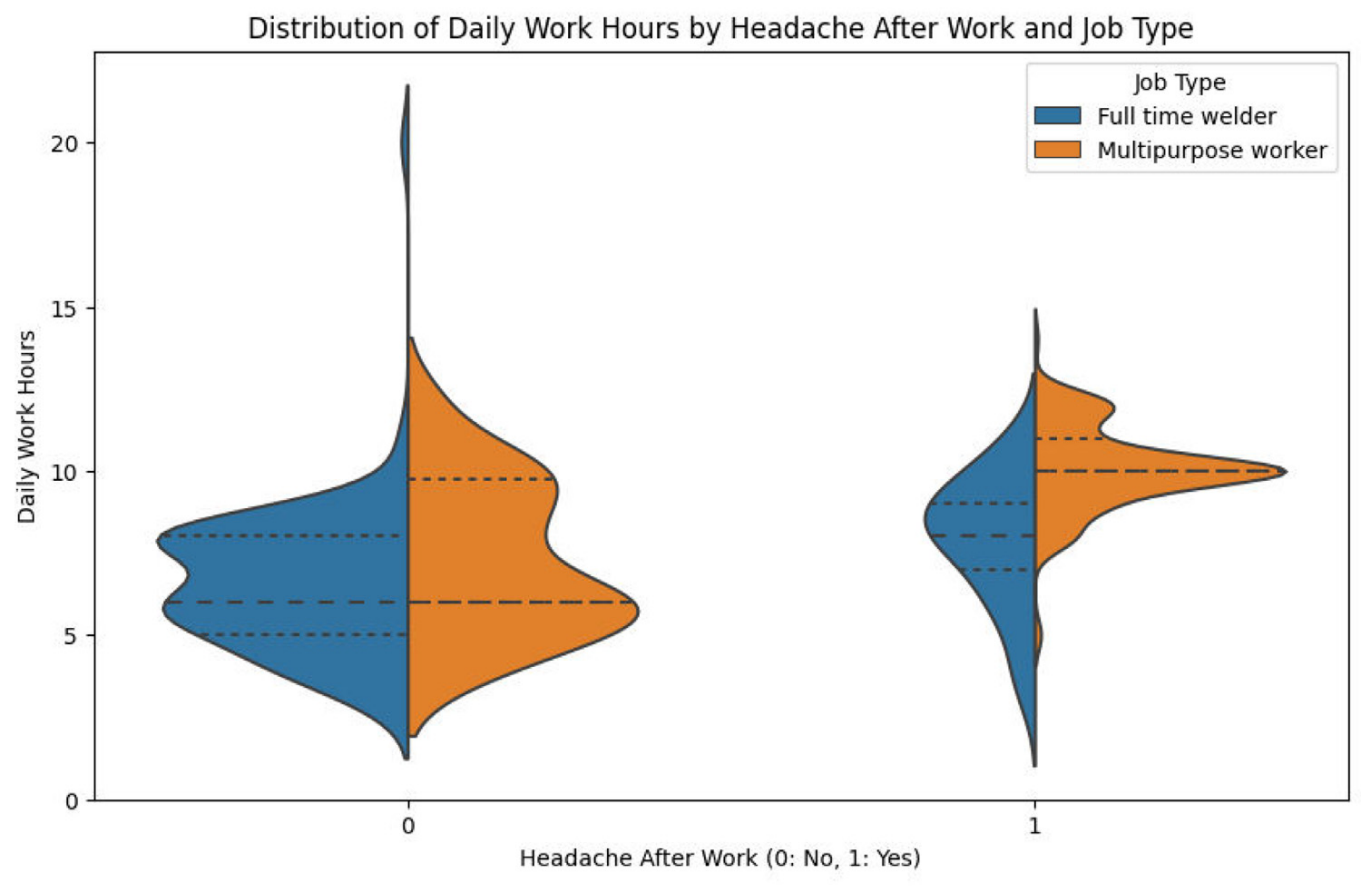
Daily work hours by headache status and job type.

Fig. 9 shows the distribution of working hours a day among welders with and without post-work headaches, divided by job type. The welders experiencing headaches worked longer on average at eight to ten hours a day, compared to five to eight hours for those who did not have headaches. Multipurpose workers would register more working hours than full-time welders, especially for those who had headaches. But there is a bit of overlap, suggesting that not all long-hour workers develop headaches, and some short-hour workers still do, suggesting that other factors such as sleep or safety procedures may also be at play.

**Fig. 10:**
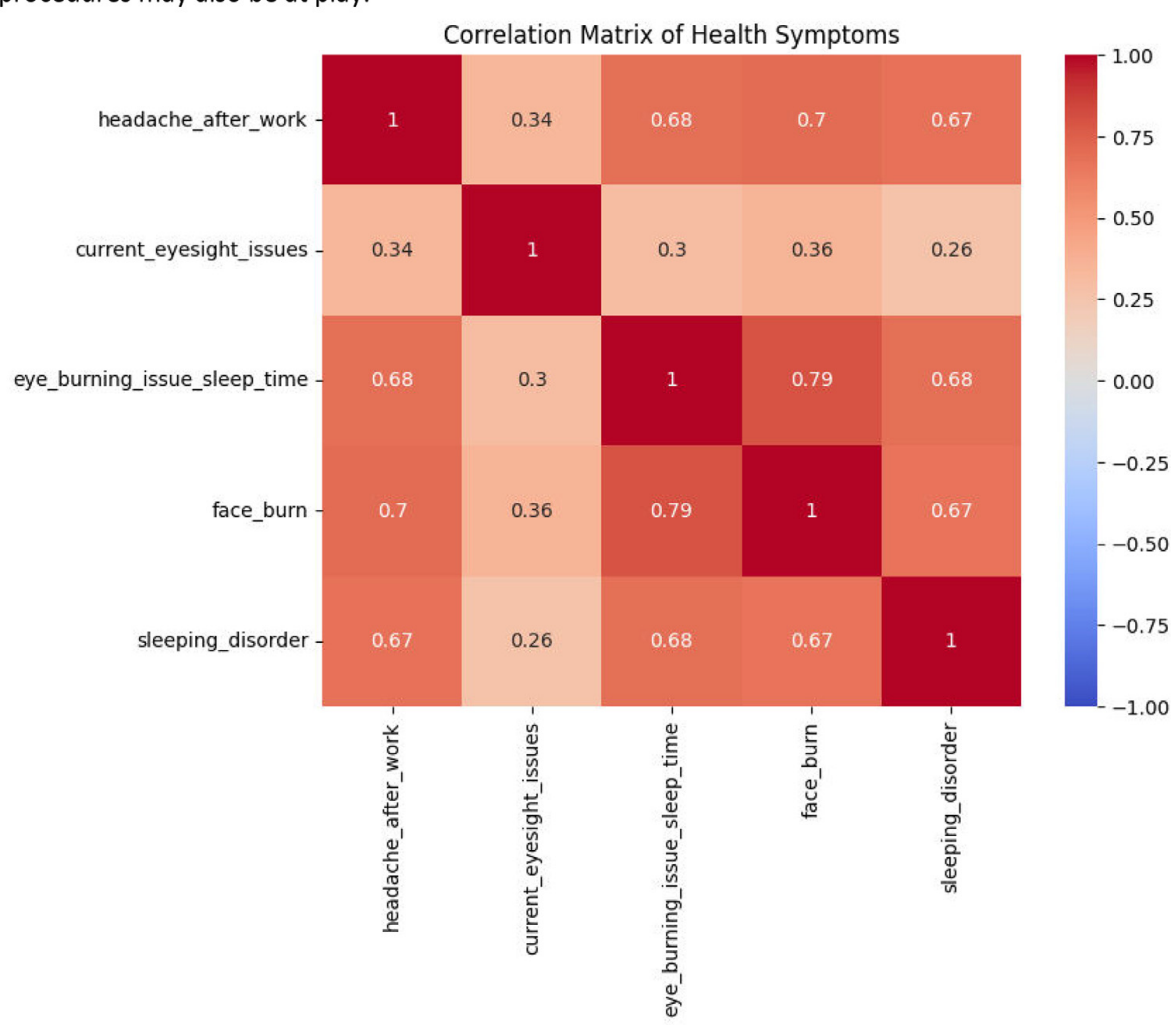
Correlations among health symptoms in welders.

Fig. 10 shows the intercorrelations of different health symptoms suffered by welders. Headache after work is positively and highly correlated with face burns, eye irritation at bedtime, and sleep disturbance, meaning these ailments are likely to occur together. Eyesight problems are less related to other health consequences. The fact that so many symptoms cluster together indicates that occupational risks may trigger a constellation of health consequences at the same time, rather than affecting welders one at a time.

## Limitations

While this dataset is significant in terms of evidence about the occupational health of welders in Bangladesh, there are a few limitations that must be kept in mind. The data, collected from 335 welders in five districts through convenience sampling, may not represent all the locations or populations of Bangladesh and therefore has limited generalizability. Because the survey was cross-sectional and relied on self-reporting health outcomes without clinician confirmation, it may have reporting biases, and causation could not be concluded. Possible confounders such as diet, environmental exposures, and ventilation in the workshop were not systematically documented, and this might impact the interpretation of health associations. Additionally, self-reported use of PPE has no observational confirmation, which may dull associations between safety interventions and health outcomes. Simple data-entry errors and subjective assessment of some health symptoms may influence accuracy. Ethical concerns, such as likely under-reporting due to a lack of formalization of work environments or fear of punishment, must be accounted for. Lastly, the predictive model presented here is a demonstration tool rather than definitive proof owing to sample size limitations and overfitting risk. Convenience/purposive sampling limits representativeness, potentially over-representing informal workshops. Random sampling across diverse settings is needed for broader generalizability.

Future research might address such limitations through nationwide, representative sampling, longitudinal follow-up, and inclusion of objective clinical assessments and other health outcomes (e.g., hearing loss, musculoskeletal disease). Additional predictive modeling and informing overall occupational health interventions in the same environments might be attained by merging this dataset with others. Convenience and purposive sampling restricts generalizability; future studies should use randomized sampling to enhance representativeness across Bangladesh.

## Ethics Statement

This study was followed by the Declaration of Helsinki and local ethical guidelines. It was a low-risk, anonymous survey of adults in a public setting, thus no formal ethical approval was required. However, each participants gave their full verbal consent, ensuring their participation was voluntary and anonymous.

## CRediT author statement

**Rakhi Moni Saha**: Data collection, curation, and writing – original draft. **Fatama Binta Rafiq**: Visualization, formal analysis. **Shimul Debnath**: Software and modeling support. **Plabon Kumar Saha**: Validation, data review. **Imran Mahmud**: Supervision, conceptualization, writing, review & editing

## Data Availability

Mendeley DataSurvey Dataset on Occupational Health, Work Conditions, and Safety Practices of Welders in Bangladesh (2022) (Original data)
